# Radiopharmaceuticals in Renal Imaging: A Comprehensive Review of Current Applications and Future Prospects

**DOI:** 10.1021/acsomega.5c08741

**Published:** 2025-12-18

**Authors:** Humeyra Battal-Kaplan, Bilge Volkan-Salancı, Suna Erdogan

**Affiliations:** † Faculty of Pharmacy, Department of Radiopharmacy, 37515 Hacettepe University, 06230 Ankara, Turkiye; ‡ Faculty of Medicine, Department of Nuclear Medicine, 37515 Hacettepe University, 06230 Ankara, Turkiye

## Abstract

The kidneys play a vital role in filtration, excretion, and metabolic regulation, making accurate functional assessment essential for diagnosis, monitoring, and management of renal diseases. While conventional imaging modalities such as ultrasonography, computed tomography (CT), and multiparametric magnetic resonance imaging (MRI) provide structural and functional information, nuclear medicine techniques enable more detailed evaluation of renal physiology at the molecular level. This review provides a comprehensive overview of functional renal imaging using both conventional renal scintigraphy and emerging positron emission tomography (PET) radiopharmaceuticals. Established renal scintigraphy radiopharmaceuticals, including ^99m^Tc-DTPA, ^99m^Tc-DMSA, ^99m^Tc-MAG3, and ^99m^Tc-EC, have long been applied to assess glomerular filtration rate (GFR) and effective renal plasma flow (ERPF). PET radiopharmaceuticals, offering higher sensitivity, superior quantification, and molecular insight, are increasingly investigated for similar purposes, with ^68^Ga-EDTA, ^68^Ga-DTPA, ^68^Ga-NOTA, ^68^Ga-DOTA, and ^18^F-FDS for GFR assessment and ^11^C-PABA, ^18^F-PFH, Re­(CO)_3_(^18^F-FEDA), and Al-^18^F-NODA-butyric acid for ERPF evaluation. Additional PET tracers, including ^68^Ga-IRDye800-tilmanocept, ^18^F-FDG, and ^68^Ga-PSMA-11, have been explored for glomerular mesangial function, renal tumors, and split renal function, respectively. By systematically summarizing both renal scintigraphy and PET approaches, this review highlights current and emerging PET radiopharmaceuticals for renal functional imaging and discusses their potential clinical applications and future perspectives in precision nephrology.

## Graphical abstract

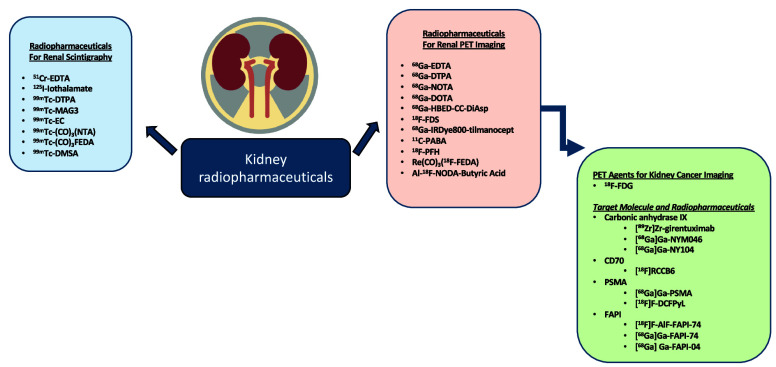

## Introduction

The kidneys are retroperitoneal organs responsible for various vital functions, including waste removal, fluid regulation, acid–base balance, and hormone secretion.^1,2^ They regulate plasma osmolarity, maintain long-term acid–base balance, produce erythropoietin to stimulate red blood cell (erythrocyte) production, secrete renin to regulate blood pressure, and convert vitamin D into its biologically active form.^3^ Consequently, accurate assessment of renal function plays a critical role in guiding clinical decision-making across a wide range of diseases.^2^

The kidney is a complex organ composed of several vascular compartments, each containing morphologically and functionally distinct cell types.^4^ Despite this complexity and heterogeneity, the clinical management of renal diseases was traditionally based on relatively limited laboratory tests. Over time, however, the need to understand disease mechanisms and progression led to the development of high-resolution imaging techniques capable of evaluating renal anatomy and function both spatially and temporally. These imaging modalities have since become integral to the study of renal physiology and pathophysiology.

Recent technological and pharmaceutical advancements have enabled clinical imaging to assess not only renal structure and morphology, but also perfusion, function, metabolism, oxygenation, and microstructure and interstitium. A wide range of imaging modalities including ultrasonography, computed tomography (CT), positron emission tomography (PET), renal scintigraphy, and multiparametric magnetic resonance imaging (MRI) are currently available to investigate the kidney at high spatial and temporal resolution. The choice of modality typically depends on the clinical question and the underlying pathological mechanism.

Tomographic imaging enables noninvasive, high-resolution differentiation of intrarenal compartments (e.g., cortex, medulla, collecting system) and allows for real-time monitoring of functional and pathophysiological processes. Conventional ultrasonography, due to its low cost, widespread availability, and safety profile, has become a standard imaging modality despite limitations in resolution and operator dependency. In the past decade, MRI has emerged as a valuable tool for characterizing renal pathophysiology. Structural and functional MRI can be combined into a single multiparametric session, enabling the assessment of renal structure, microstructure, and functional heterogeneity. Moreover, the absence of ionizing radiation makes MRI particularly suitable for serial imaging, even in patients with impaired renal function, especially pediatric patients.^5^

For the evaluation of kidney function, parameters such as renal perfusion and clearance are essential, and nuclear medicine offers the advantage of quantifying both. Functional imaging techniques provide dynamic data, revealing not only perfusion but also renal clearance and excretion patterns. Moreover, radiopharmaceutical-based imaging enables the extraction of semiquantitative and quantitative parameters, contributing to improved diagnosis and patient monitoring.

Functional imaging offers noninvasive evaluation of renal function. Thanks to advancements in technology, the development of new radiopharmaceuticals, and simplified imaging protocols, clinicians can now obtain fast, accurate, and reproducible diagnostic information.^6^ Radiopharmaceuticals for renal scintigraphy have been in clinical use for a long time.^1,7,8^

PET, another imaging modality, is a powerful molecular imaging tool with broad applications, including disease diagnosis, treatment response monitoring, and early phase assessment of the pharmacokinetics and pharmacodynamics of new therapeutic agents.^9^ PET offers superior spatial and temporal resolution, enabling dynamic three-dimensional imaging.^2^ Compared to scintigraphy, PET provides higher count rates, allowing for the use of lower radiotracer doses. This not only reduces radiation exposure but also improves quantitative accuracy, making PET particularly advantageous for pediatric imaging.^8,10^

These advantages have significantly increased interest in PET imaging and PET-based radiopharmaceuticals.^11,12^ By combining PET with CT, clinicians can simultaneously obtain detailed anatomical and functional information, facilitating comprehensive evaluation of renal function.^1^ In recent years, numerous studies have focused on the development of novel PET radiopharmaceuticals specifically designed for assessing renal function.^8^

This review provides an overview of both clinically established and newly developed radiopharmaceuticals that are advantageous for evaluating renal function.

## 1 Radiopharmaceuticals for Renal Scintigraphy

Major advancements in noninvasive imaging techniques, particularly over the past decade, have provided significant advantages in visualizing biological processes for medical diagnosis. Scintigraphy is a functional imaging modality that enables visualization by detecting γ rays emitted from radionuclides. Commonly used radionuclides in this context include technetium-99m (Tc-99m), indium-111 (In-111), gallium-67 (Ga-67), and iodine-123 (I-123).^13^

Among these, Tc-99m is the most widely used radionuclide in clinical practice due to its generator-based availability, optimal half-life, and favorable chemical properties. It emits 140 keV γ photons, which provide high-efficiency imaging with relatively low radiation exposure to patients. Furthermore, the development of cold kits for the simple preparation of Tc-99m-labeled radiopharmaceuticals represents a major advantage, contributing to its widespread clinical use.^14,15^ Because of these favorable characteristics, Tc-99m remains indispensable in diagnostic nuclear medicine.^16^

In nuclear medicine imaging, the assessment of renal function includes both *in vivo* and *in vitro* methods and involves both static and dynamic imaging modalities, as illustrated in Figure 1. Static imaging provides information on renal morphology, cortical anatomy, and the relative distribution of function, while dynamic imaging allows for evaluation of renal perfusion, extraction, and excretion processes.

**Figure 1:**
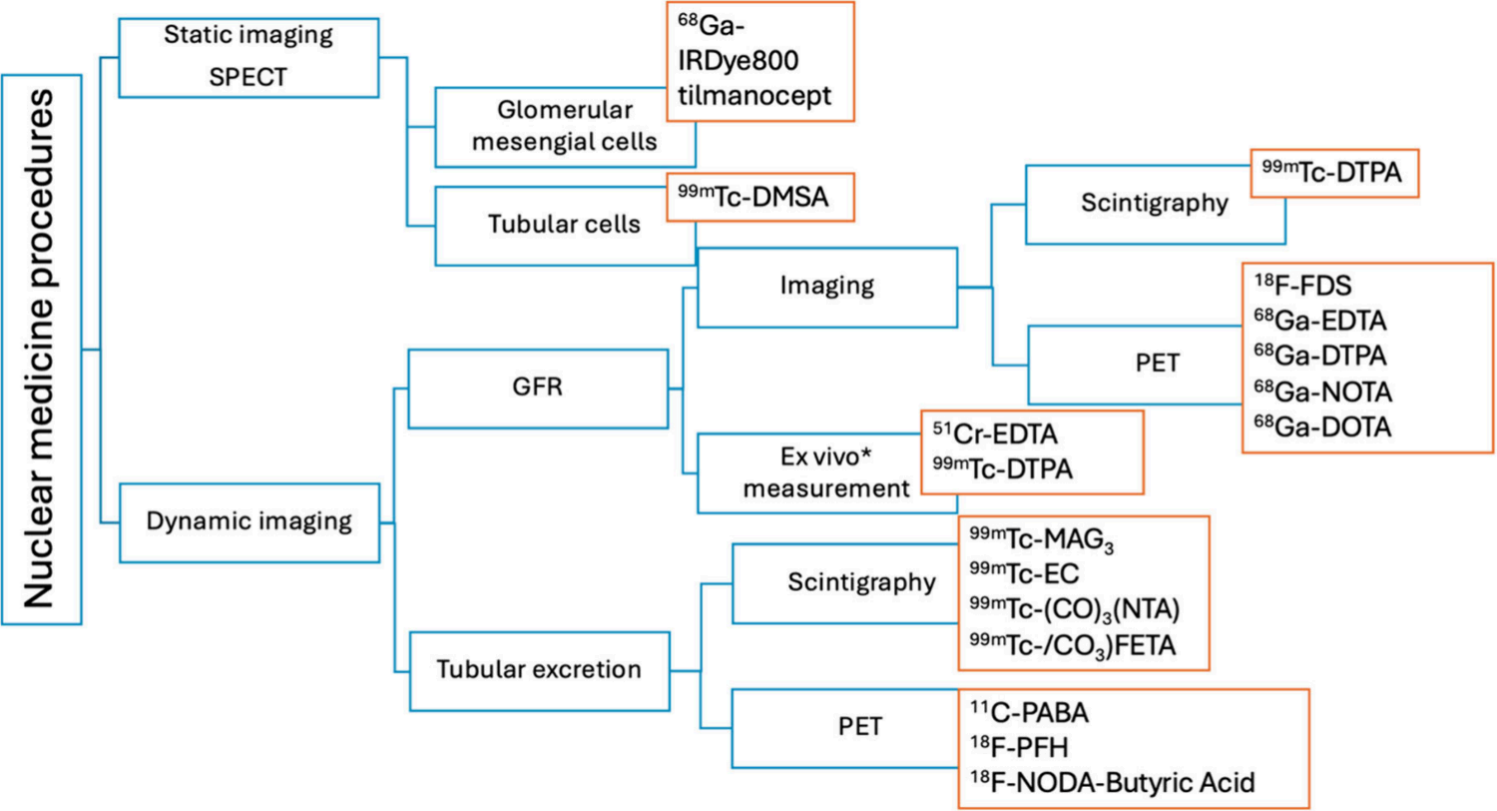
Radiopharmaceuticals used for renal functions.

The first radiopharmaceutical used for renal imaging was radioactive mercury (^197^Hg or ^203^Hg)-labeled chlormerodrine, introduced in the 1960s. This agent is taken up by renal tubular cells and trapped in cellular cytoplasm. Subsequently, from the mid-1960s to the mid-1980s, iodine-131-labeled orthoiodohippurate (^131^I-OIH) was commonly used in renal scintigraphy.^17^ Although ^131^I-OIH proved useful, particularly in patients with poor renal function and in complex data analysis, its use was limited by several disadvantages. These include high radiation dose, especially in the presence of obstructive uropathy, poor spatial resolution, limited sensitivity, high photon energy incompatible with conventional γ cameras, and restricted allowable administered dose.

To address these issues, ^123^I-labeled iodohippurate was developed as an alternative. Iodine-123 exhibits more favorable nuclear characteristics, including a γ photon energy of 159 keV and a half-life of 13.2 h. However, it is cyclotron-produced, making it relatively more expensive. Furthermore, it may contain traces of iodine-124 (I-124), which can increase radiation dose and reduce image quality if produced via low-energy cyclotron reactions. As a result, its routine clinical use has remained limited.

Following the 1970s, Tc-99m-labeled renal radiopharmaceuticals were introduced into clinical practice and have since gained substantial importance in renal imaging.^18^

Radiopharmaceuticals used in renal scintigraphy are typically classified into three functional categories:^1,19^

1. Agents filtered by the glomeruli—used for measuring the glomerular filtration rate (GFR).
2. Agents secreted by renal tubules via organic anion transporters—used for evaluating effective renal plasma flow (ERPF).
3. Agents retained in renal tubules through receptor-mediated endocytosis—used in static renal scintigraphy or single-photon emission computed tomography (SPECT) for anatomical and functional assessment.

### 1.1 Glomerular Filtration Rate (GFR) Measurement

The glomerular filtration rate (GFR), widely regarded as the most reliable indicator of kidney function, is defined as the volume of plasma filtered by the glomeruli per unit of time.^20^ Glomerular filtration is a passive process and can be estimated by measuring the rate at which a substance is cleared from the plasma. In adults, the GFR is approximately 50–60 mL/min for each kidney. In a normally hydrated individual, only about 1% of the glomerular filtrate reaches the collecting system; the remaining 99% is reabsorbed by the tubules and returned to the bloodstream.^21^ Accurate determination of GFR is a critical component in evaluating renal function.^22^

However, because the filtration process occurs simultaneously in millions of glomeruli, direct measurement of GFR is not feasible in clinical research settings. Additionally, the composition and volume of the filtrate change as it passes through the nephron.^2,10,23^ Therefore, GFR is typically estimated using exogenous or endogenous filtration markers.^24^

Inulin is a fructose polymer with a molecular weight of approximately 5000 and an inert molecule that is freely filtered by the glomeruli and neither secreted nor reabsorbed by the tubules. It is considered the nonradioactive gold standard for measuring GFR.^25,26^ Although inulin clearance provides the best measure of GFR, it is not routinely used in clinical practice due to the complexity and cost of test. This limitation has led to the search for alternative markers that allow for simpler and more practical estimation of GFR.

Currently, GFR is most commonly estimated using serum creatinine concentration.^8,24^ Although this method is convenient and widely available, it lacks the sensitivity to detect mild or early changes in GFR.^27^ Furthermore, since creatinine is partially secreted by the proximal tubules, this can result in an overestimation of GFR, leading to potential misinterpretation. Another limitation of this method is its inability to assess split renal function, which is often necessary in various clinical scenarios.^8^

Several exogenous markers have been proposed as alternatives to improve accuracy, including the radioactive tracers chromium-51 ethylenediaminetetraacetic acid (^51^Cr-EDTA), iodine-125 iothalamate (^125^I-iothalamate), and technetium-99m diethylenetriaminepentaacetic acid (^99m^Tc-DTPA).

#### ^51^Cr-Ethylenediaminetetraacetic Acid (^51^Cr-EDTA)

Chromium-51 (^51^Cr) is a synthetic radioactive isotope of chromium with a half-life of 27.7 days that decays via electron capture, emitting γ rays of 0.32 MeV.

EDTA, a widely used chelating agent, forms stable complexes with metal ions, including radionuclides, due to its multidentate structure, which allows coordination through multiple binding sites such as amine and carboxylate groups. The two amine and four carboxylate protonation sites in EDTA provide stable coordination with metal ions, and these complexes remain intact in biological systems.^28–30^ The physiologically stable chelator EDTA and its radiolabeled form, ^51^Cr-EDTA, are used for monitoring glomerular filtration and quantitatively assessing glomerular filtration rate (GFR), as they are eliminated exclusively via glomerular filtration and exhibit clearance properties similar to inulin.^31,32^ Introduced by Garnett et al. approximately 30 years ago,^33^ ^51^Cr-EDTA has been widely accepted as a simple, safe, and reliable method for renal function assessment. GFR determination using ^51^Cr-EDTA is typically performed by constructing time–activity curves derived from multiple blood samples collected after a single intravenous injection of the tracer. The total area under the plasma elimination curve (AUC) is used to calculate GFR, under the assumption that total plasma clearance reflects renal clearance alone. Although more invasive and costly than inulin clearance, ^51^Cr-EDTA demonstrates strong correlation with inulin-based measurements and is considered the gold standard for routine clinical GFR assessment.^34,35^

Nonetheless, limitations include its inability to provide split renal function, the requirement for multiple blood samples, and occasional issues with radiopharmaceutical availability.^8,36^ Furthermore, studies indicate that ^51^Cr-EDTA may have significant extrarenal clearance; Moore et al. reported that conventional GFR measurements using ^51^Cr-EDTA overestimate true renal clearance by approximately 10%, likely due to the plasma clearance curve not reaching the true terminal exponential phase by 2 h postinjection.^37^

#### Iodine-125-Labeled Iothalamate (^125^I-Iothalamate)

^125^I-iothalamate is cleared from the body exclusively by glomerular filtration, without undergoing tubular secretion or reabsorption. Its renal clearance has been reported to be statistically comparable to that of inulin; therefore, it has been utilized for GFR measurement using plasma sampling techniques.^37^ However, the absence of high-energy photon emission suitable for imaging and the relatively long half-life of approximately 60 days have limited the clinical use of ^125^I-iothalamate.^38^

#### ^99m^Tc-Diethylenetriaminepentaacetic Acid (^99m^Tc- DTPA)

^99m^Tc-DTPA is minimally bound to plasma proteins (5–10%), diffuses freely into the extravascular space, and is filtered exclusively by the glomeruli without undergoing tubular secretion or reabsorption.^39^ Due to these properties, it has been recommended as a rapid, noninvasive, and reliable option for measuring glomerular filtration rate (GFR).^40^ The clearance of ^99m^Tc-DTPA has been shown to correlate well with that of ^51^Cr-EDTA,^32^ and it has thus been developed as an alternative radiopharmaceutical for GFR assessment.

In a multicenter prospective study comparing ^99m^Tc-DTPA and ^51^Cr-EDTA, both radiopharmaceuticals were administered simultaneously to 88 patients. Urine and plasma clearances, as well as volumes of distribution, were evaluated. The results showed urinary clearances of 64.1 ± 27.6 mL/min for ^51^Cr-EDTA and 66.1 ± 28.0 mL/min for ^99m^Tc-DTPA. Plasma clearances were 66.1 ± 25.8 mL/min for ^51^Cr-EDTA and 68.1 ± 26.6 mL/min for ^99m^Tc-DTPA. The distribution volumes were reported as 17.3 ± 4.6 L for ^51^Cr-EDTA and 16.6 ± 4.6 L for ^99m^Tc-DTPA. These findings suggest that the high accuracy and precision of ^99m^Tc-DTPA make it a suitable alternative to ^51^Cr-EDTA in GFR measurement.^36^

In addition, several studies have demonstrated the utility of ^99m^Tc-DTPA renal dynamic scintigraphy in the evaluation of renal function, particularly in patients with suspected renal failure, and in the investigation of extrarenal abnormalities through radionuclide uptake in pathological lesions.^39,41^ However, its renal extraction fraction is approximately 20% at 30 min, which limits its effectiveness in patients with significantly impaired renal function. Therefore, its use is not recommended in cases where urinary tract obstruction is suspected.^42^

Despite these limitations, ^99m^Tc-DTPA offers several advantages: it is inexpensive, widely available, and associated with a low radiation dose to the patient. Moreover, it is suitable for γ camera imaging, allowing for the simultaneous acquisition of a renogram and calculation of differential renal function (Figure 2).

**Figure 2:**
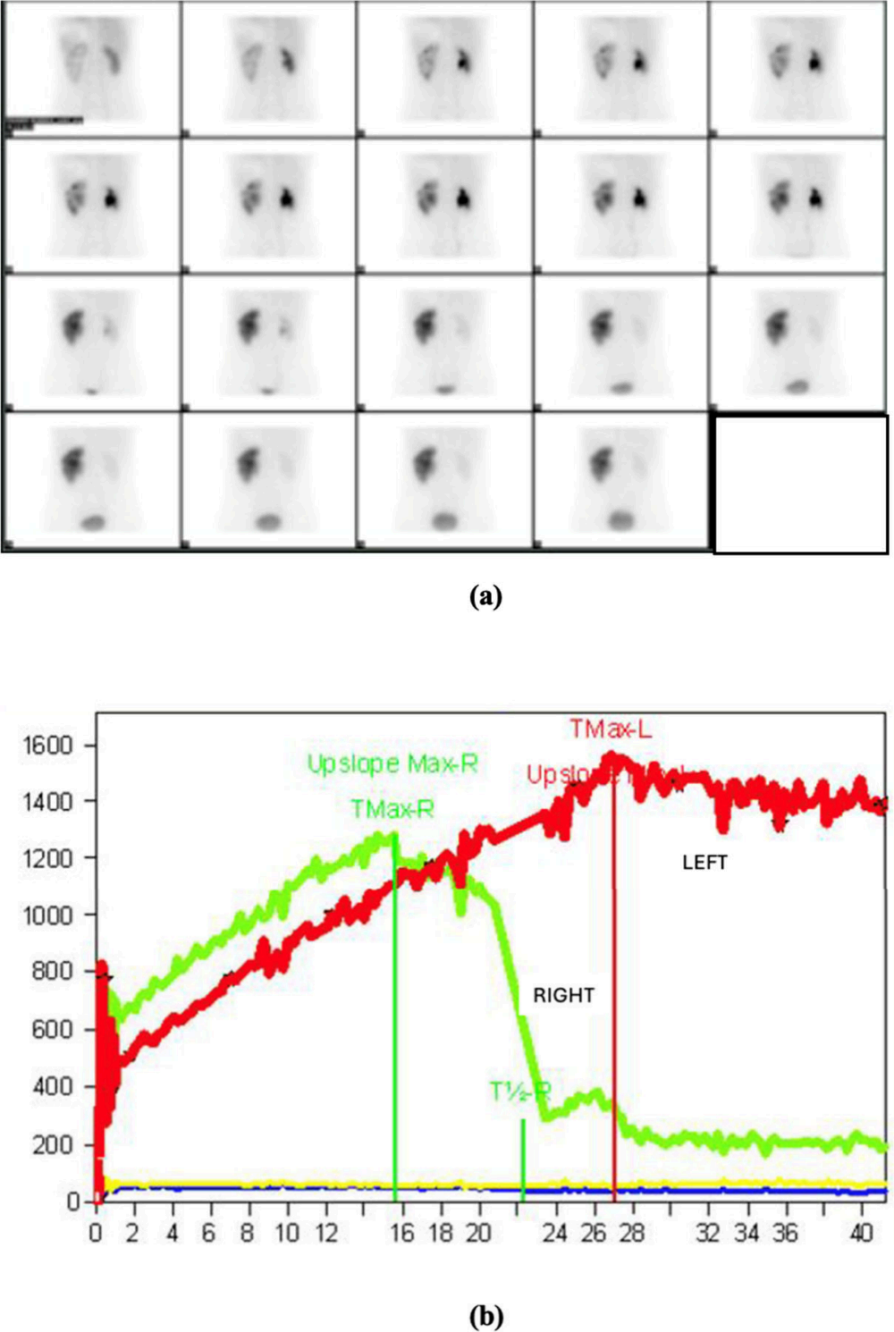
(a) ^99m^Tc-DTPA scintigraphy dynamic images and (b) time–activity curves of the kidneys. The image is from the authors’ clinical database.

### 1.2 Effective Renal Plasma Flow (ERPF) Measurement

Renal plasma flow (RPF), also referred to as effective renal plasma flow (ERPF), is a fundamental indicator of renal function that reflects the kidneys’ capacity to filter waste products from the bloodstream. It represents the volume of plasma from which a given substance is completely cleared by the kidneys per unit time. One of the main limitations of this measurement is that a reduction in the secretory capacity of the renal tubules can lead to a significant underestimation of ERPF.^43^ Moreover, several factors including age, sex, underlying kidney disease, and the use of certain medications, such as nonsteroidal anti-inflammatory drugs (NSAIDs), can influence ERP.

Analogous to the use of inulin clearance as the gold standard for GFR measurement, *p*-aminohippuric acid (PAH) clearance is considered the gold standard for assessing ERPF. However, PAH clearance is not routinely employed in clinical practice due to the lengthy and complex chemical analysis it requires. Additionally, PAH does not provide information about individual kidneys or their compartments.

#### *p*-Aminohippuric Acid (PAH)

*p*-Aminohippurate (PAH), also known as *p*-aminohippuric acid, is a urinary metabolite that does not bind to plasma proteins and is impermeable to the erythrocyte membrane. As an anionic substrate, PAH is frequently used to evaluate RPF and GFR. At low concentrations, PAH is almost completely cleared from plasma through glomerular filtration and active tubular secretion during a single renal pass. In individuals with normal renal function, the urinary PAH extraction ratio is approximately 0.92. Based on this high extraction efficiency, urinary clearance of PAH can be used to provide an accurate estimation of ERPF.^44^

The RPF calculation using the clearance of *p*-aminohippuric acid (PAH) is done with the following formula:^45^

$$\mathrm{RPF}=C_{\mathrm{PAH}}=U_{\mathrm{PAH}}\times V/P_{\mathrm{PAH}}$$

where RPF is renal plasma flow (mL/min or mL/24h), *C*_PAH_ is the PAH clearance (mL/min or mL/24 h), *U*_PAH_ is the PAH urine concentration (mg/mL), *V* is the urine flow rate (mL/min or mL/24 h), and *P*_PAH_ is the PAH plasma concentration (mg/mL).

#### ^99m^Tc-Mercaptoacetyltriglycine (^99m^Tc-MAG3)

^99m^Tc-MAG3 is a radiopharmaceutical commonly used in dynamic renal imaging. It is actively secreted by the renal tubules and is employed primarily for the estimation of ERPF.^46^ ^99m^Tc-MAG3 binds to plasma proteins in a highly reversible manner, with a binding rate of approximately 79–90%.^47^ Its rapid renal clearance makes it particularly useful for evaluating renal function in cases where GFR is low.^42^

This radiopharmaceutical is considered the agent of choice for assessing renal transplants, diagnosing acute tubular necrosis, and conducting tubular function scintigraphy. The extraction fraction of ^99m^Tc-MAG3 is significantly higher than that of ^99m^Tc-DTPA (20%), which allows for superior diagnostic performance, especially in adult and pediatric patients with suspected urinary tract obstruction.^48^

In a study by Lim and Choi,^47^ the ability of ^99m^Tc-MAG3 to differentiate between obstructed and nonobstructed kidneys was investigated using a rabbit model. Renal scintigraphy was performed to assess individual kidney function, and findings were corroborated with pathological and morphological examinations. The study concluded that renal size and morphology could be effectively visualized with ^99m^Tc-MAG3 scintigraphy, and that the method was capable of distinguishing obstructive from nonobstructive renal conditions.

Additionally, ^99m^Tc-MAG3 has been reported as a viable alternative to ^99m^Tc-dimercaptosuccinic acid (^99m^Tc-DMSA), particularly for detecting renal abnormalities in pediatric patients.^47^ In a retrospective study, renal cortical evaluation and split renal function (SRF) measurements obtained via ^99m^Tc-MAG3 scintigraphy were found to be comparable to those acquired with ^99m^Tc-DMSA. The study also emphasized that ^99m^Tc-MAG3 offers important insights into the urodynamic status of both kidneys, reduces radiation exposure in children, and shortens imaging time, making it advantageous in pediatric clinical practice.^49^

#### ^99m^Tc-Ethylene Cysteine (^99m^Tc-EC)

^99m^Tc-EC is a radiopharmaceutical derived from the metabolite of ethylene cysteine dimer (ECD) and is considered an alternative to ortho-iodohippurate. It serves as a marker of renal tubular function and exhibits imaging characteristics similar to those of ^99m^Tc-MAG3.^50^ Following intravenous administration, a portion of ^99m^Tc-EC is secreted by organic anion transporters located in the proximal tubules. The compound exhibits 30% plasma protein binding and has a renal extraction fraction of 0.70, which is higher than that of ^99m^Tc-MAG3. It is predominantly excreted via the urinary system and demonstrates minimal hepatic uptake compared to ^99m^Tc-MAG3.^42^ Approximately 70% of the administered dose is excreted within 40 min, and up to 95% is eliminated within 1.5 h after injection.^51^

In a clinical study involving 35 patients, ^99m^Tc-EC was compared with ^99m^Tc-MAG3 for the evaluation of renal function. The findings indicated that renal uptake of ^99m^Tc-EC was significantly higher, and hepatic activity was significantly lower, than that observed with ^99m^Tc-MAG3.^52^

Another study compared kidney function measurements obtained through dynamic scintigraphy using ^99m^Tc-EC and ^99m^Tc-DTPA with those obtained from static scintigraphy using ^99m^Tc-DMSA. The results showed that relative renal function assessed with ^99m^Tc-EC was comparable to the measurements obtained with ^99m^Tc-DMSA. In contrast, relative function values derived from ^99m^Tc-DTPA differed significantly from those of ^99m^Tc-DMSA.^53^

#### ^99m^Tc-(CO)_3_-Tricarbonyl Nitrile Acetic Acid [^99m^Tc-(CO)_3_(NTA)]

A small proportion of ^99m^Tc-MAG3, a radiopharmaceutical frequently used for the measurement of ERPF, is excreted via the hepatobiliary system, and this excretion rate increases as renal function declines. This limitation prompted researchers to investigate alternative radiopharmaceuticals that could yield more accurate results under such conditions. Among these, ^99m^Tc-(CO)_3_(NTA) has been the subject of several studies. This compound is a stable dianionic complex at physiological pH, and the presence of a carboxylate group facilitates tubular transport.^54^

The performance of ^99m^Tc-(CO)_3_(NTA) was compared with that of ^99m^Tc-MAG3 in patients with suspected urinary tract obstruction. The clearance of ^99m^Tc-(CO)_3_(NTA) was significantly higher (331 ± 146 mL/min/1.73 m^2^) than that of ^99m^Tc-MAG3 (271 ± 105 mL/min/1.73 m^2^), and the kidney-to-background activity ratio was also superior for ^99m^Tc-(CO)_3_(NTA). Importantly, no gallbladder activity or hepatobiliary excretion was observed with ^99m^Tc-(CO)_3_(NTA). Based on the study results, it was concluded that obstructive findings not clearly identified with ^99m^Tc-MAG3 could be better delineated using NTA images and renograms.^55^

Furthermore, studies have demonstrated that the pharmacokinetic properties of ^99m^Tc-(CO)_3_(NTA) are comparable to those of ^131^I-OIH, which is considered a reference standard for ERPF measurement.^56,57^ In a study conducted in healthy volunteers, the plasma clearance values of ^99m^Tc-(CO)_3_(NTA) and ^131^I-OIH were found to be nearly identical (475 ± 105 mL/min vs 472 ± 108 mL/min, respectively). Additionally, ^99m^Tc-(CO)_3_(NTA) exhibited significantly lower binding to plasma proteins and erythrocytes compared to ^131^I-OIH. The study also reported high-quality imaging and renogram parameters comparable to those obtained with ^131^I-OIH.^57^

#### ^99m^Tc-(CO)_3_ *N*-(Fluoroethyl)­iminodiacetic Acid [^99m^Tc-(CO)_3_FEDA]

^99m^Tc-(CO)_3_FEDA is a stable and well-defined tricarbonyl complex containing an uncharged fluoroethyl side group. Because of its rapid renal clearance, it demonstrates renal tracer characteristics comparable to those of ^131^I-OIH.^58^

Studies have shown that ^99m^Tc-(CO)_3_FEDA can be synthesized with high radiochemical purity (>99%) and maintains its stability for up to 24 h postsynthesis.^58,59^ To assess the importance of a negatively charged uncoordinated carboxyl group for OAT1 transporter recognition and tubular secretion, Lipowska et al. evaluated the pharmacokinetics of three new monoanionic ^99m^Tc­(CO)_3_(NTA) analogs (coded as ADA, HAD and FEDA) with uncharged pendant groups but with inner coordination spheres identical to that in ^99m^Tc­(CO)_3_(NTA). All compounds are rapidly eliminated from the bloodstream and exhibits high specificity for renal excretion. Activity in the urine, as a percent of ^131^I-OIH at 10 and 60 min, was 96% and 99% for ADA, 96% and 100% for HDA, and 100% and 99% for FEDA, respectively. Each new tracer was excreted unchanged in the urine. Furthermore, its pharmacokinetic profile has been reported to be comparable to that of both ^99m^Tc-(CO)_3_(NTA) and ^131^I-OIH, suggesting its potential as an effective radiopharmaceutical for the evaluation of renal function.^59^

### 1.3 Static Kidney Scintigraphy–Renal Cortical Imaging

#### ^99m^Tc-Dimercaptosuccinic Acid (^99m^Tc-DMSA)

^99m^Tc-DMSA is a static imaging agent that binds primarily to plasma proteins, particularly α1-microglobulin and this protein bound DMSA complex is filtered through the glomerulus and subsequently taken up by proximal tubular cells via megalin/cubilin receptor-mediated endocytosis, where it becomes trapped in the mitochondrial compartment. Consequently, ^99m^Tc-DMSA serves as a marker of proximal tubular endocytic activity.^60^ Once internalized, it remains bound to the renal cortical tubules for at least 6 h.^61^

Its primary clinical application is in the assessment of cortical anatomy and the detection of structural abnormalities, such as renal ectopia or renal scarring (Figure 3). In particular, ^99m^Tc-DMSA enables the identification of renal scars following episodes of acute pyelonephritis.^5^ Recurrent urinary tract infections (UTIs) are a major contributor to acquired and permanent renal parenchymal damage, especially in the pediatric population. For this purpose, ^99m^Tc-DMSA cortical kidney scintigraphy (CKS) has long been employed as a minimally invasive diagnostic tool with high sensitivity (96%) and specificity (98%) for detecting renal damage secondary to pyelonephritis in children.^62^ Moreover, several studies have demonstrated that ^99m^Tc-DMSA scintigraphy not only confirms the presence of renal damage but also enables quantification of the extent of injury.^63^

**Figure 3:**
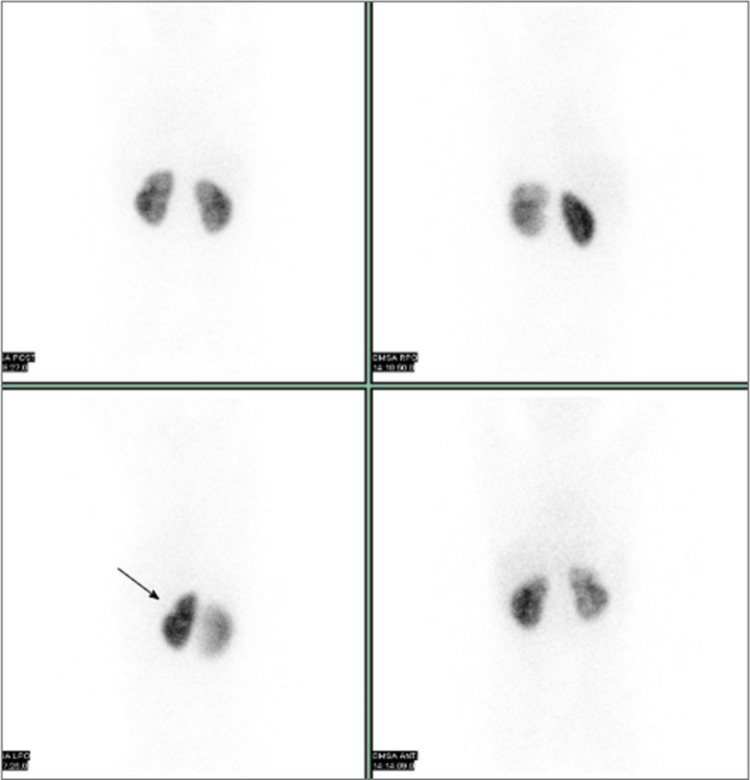
^99m^Tc-DMSA scintigraphy of a 10-year-old girl. A parenchymal defect is observed on the left kidney secondary to acute pyelonephritis (arrow). The image is from the authors’ clinical database.

In a comparative study by Momin et al.,^64^ relative renal function (RRF) calculated using ^99m^Tc-DTPA and ^99m^Tc-DMSA was evaluated in 50 patients aged 5 months to 72 years. The findings revealed that both radiopharmaceuticals produced comparable RRF values. While ^99m^Tc-DMSA may be considered the preferred agent for the assessment of RRF due to its cortical specificity, ^99m^Tc-DTPA was suggested to be more suitable when GFR estimation and renogram curve analysis are required.

## 2 Renal PET Radiopharmaceuticals

Although kidney imaging has been performed using scintigraphy for many years, PET imaging offers several advantages over γ-camera-based methods, particularly in oncology, including kidney imaging.^46^ These advantages include higher sensitivity, improved signal-to-background ratio, and the capacity to handle higher counting rates. Additionally, the simultaneous detection of two annihilation photons by PET detectors enables better image resolution and more accurate quantification. PET also allows for the administration of lower doses of radioactivity while providing more precise measurements of regional radiopharmaceutical concentrations in tissues.^65^ This represents a significant advantage in minimizing radiation exposure, especially in infants and children.

Furthermore, with the development of new PET agents, it is now possible to obtain information not only about the current functional status of the kidneys but also about various pathophysiological processes within the renal parenchyma. These include inflammation, renal blood flow (RBF), overexpression of angiotensin II type 1 receptors (AT1R), mitochondrial complex I (MC-I) activity, and kidney cancer.^46^ As a result, recent years have seen increased efforts to develop novel PET radiopharmaceuticals for the assessment of kidney function.^8^

Fluorine-18 (F-18) and gallium-68 (Ga-68) are among the most commonly used radionuclides for the production of PET radiopharmaceuticals.^66^ F-18 has a half-life of 110 min, a maximum positron energy of 0.635 MeV, and is typically produced in a cyclotron via the ^18^O­(p,n)^18^F reaction.^67^ Because of its favorable nuclear and physical characteristics—including a high positron decay ratio (97%), appropriate half-life for clinical use, and low positron energy—F-18 has attracted considerable attention in radiochemistry. Radiotracers labeled with F-18 offer excellent image quality with lower radiation doses, due to its high positron yield and low positron energy.^8^

Ga-68, on the other hand, is a generator-produced radionuclide with a shorter half-life of 68 min and a positron energy of 1.90 MeV.^68^ Its short half-life results in lower radiation exposure for patients, while its high positron decay rate contributes to high-quality imaging. These properties make Ga-68 a highly suitable radionuclide for diagnostic imaging.^31^

### 2.1 PET Radiopharmaceuticals Used for GFR Assessment

^99m^Tc-DTPA is the most commonly used radiopharmaceutical for evaluating renal function in clinical practice; however, it has several limitations. For instance, its reliability may be compromised in patients with renal abnormalities, excessive body weight, or in pediatric populations. In addition, planar imaging provides limited spatial information, particularly in patients with space-occupying renal lesions, which may result in inaccurate assessments, the need for repeat examinations, and increased healthcare costs.

PET imaging systems offer numerous advantages over traditional γ-camera-based renal imaging. These include superior spatial and temporal resolution, higher sensitivity, accurate and absolute camera-based quantification, dynamic 3D tomographic imaging, anatomical coregistration with CT, and enhanced image quality at lower radiation doses. Given these advantages, PET imaging presents a promising noninvasive alternative for GFR assessment. Its ability to produce high-quality images with reduced radioactivity dose also has important implications for improving patient safety.^69^

Physiologically stable metal chelates such as EDTA and DTPA are excreted by the kidneys via glomerular filtration and have been widely investigated for monitoring renal function and quantitatively assessing GFR. Accordingly, these agents have been labeled with PET tracers, in addition to traditional SPECT tracers, for GFR evaluation.^31^ Moreover, the potential utility of other chelators, such as NOTA and DOTA, has also been explored for this purpose.

#### ^68^Ga-Ethylenediaminetetraacetic acid (^68^Ga-EDTA)

EDTA is a well-known chelating agent with a strong capacity to form stable complexes with most metal ions and is completely filtered by the renal glomeruli. An ideal GFR tracer should be exclusively removed from the plasma by glomerular filtration and should not bind to blood components. EDTA exhibits low plasma protein binding, which facilitates its rapid and specific clearance through glomerular filtration (Figure 4).

**Figure 4:**
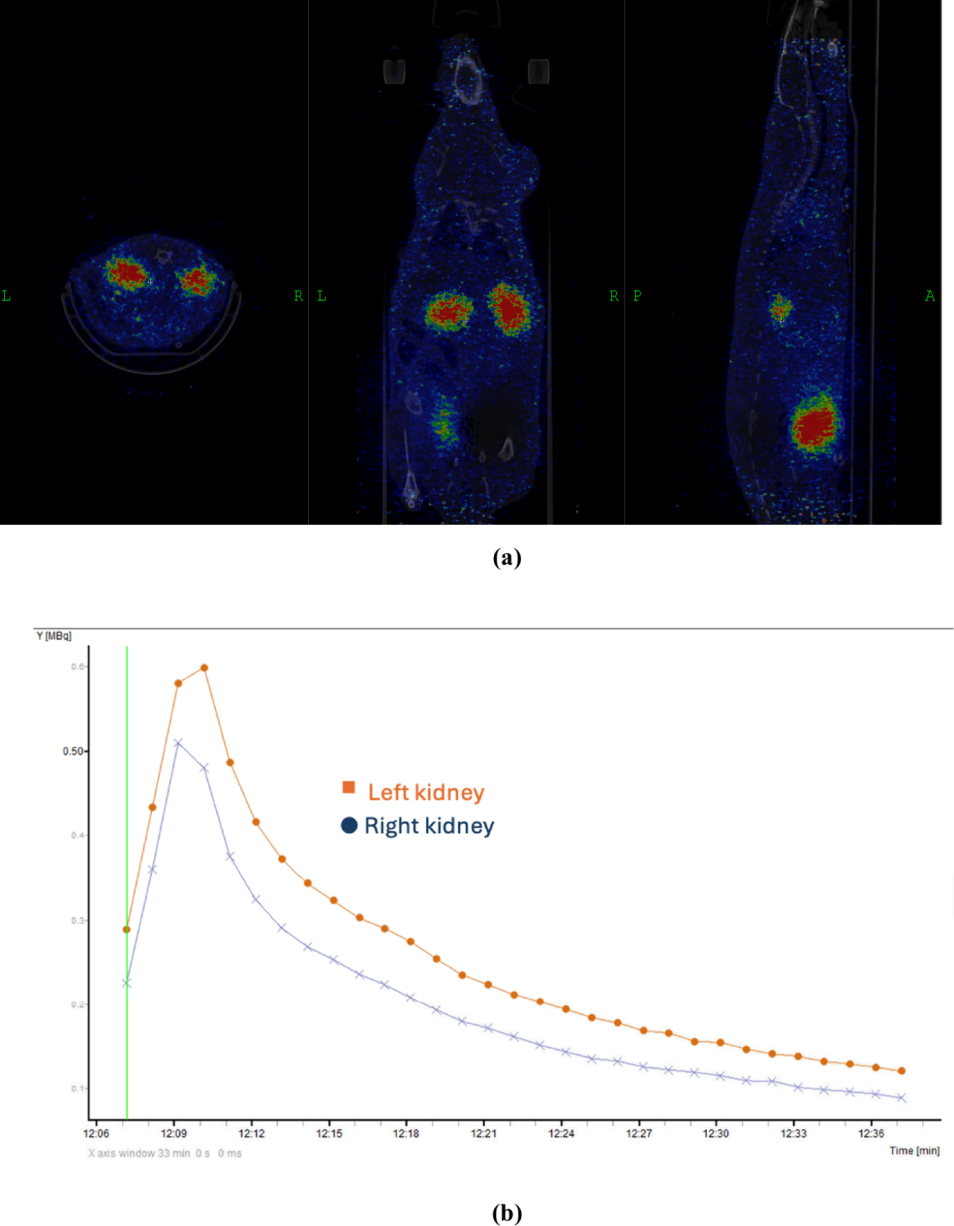
(a) Whole-body ^68^Ga-EDTA PET images of a wistar rat (male, approximately 300 g), acquired 30 min after i.v. injection, and (b) time–activity curves of the kidneys derived from dynamic Ga-68 EDTA PET acquisition. (Original work of the authors.)

The first study demonstrating the use of ^68^Ga-EDTA for measuring blood volume and GFR was conducted by Yamashita et al. in 1988. The results obtained from both young and elderly subjects were consistent with previous findings in the literature, suggesting that ^68^Ga-EDTA may be a highly applicable radiopharmaceutical for quantitative GFR assessment.^70^

In a subsequent study comparing ^51^Cr-EDTA, the gold standard for GFR measurement, with ^68^Ga-EDTA, it was shown that the results obtained using ^68^Ga-EDTA were in agreement with those of ^51^Cr-EDTA.^71^ Although studies on ^68^Ga-EDTA remain limited, the findings reported so far are promising.^31,69,72,73^ These studies demonstrate that ^68^Ga-EDTA could serve as an alternative to ^99m^Tc-DTPA, the most widely used SPECT agent in current clinical practice, for several clinical indications.^31^

A recent study conducted in mouse models with acute kidney injury and unilateral ureteral obstruction further investigated the utility of ^68^Ga-EDTA in renal imaging. The study highlighted its potential as an ideal and reliable agent for assessing kidney function in preclinical models of renal damage.^69^

#### ^68^Ga-Diethylenetriaminepentaacetic Acid (^68^Ga-DTPA)

DTPA is a metal chelator that, like EDTA, is filtered by the glomeruli.^46,74^ It has been successfully used for many years in renal imaging and for the evaluation of GFR through labeling with Tc-99m. Current studies are investigating whether ^68^Ga-DTPA can be employed in a similar manner.^75,76^

In a related study, the binding affinities of ^68^Ga-labeled DTPA and EDTA to plasma proteins and blood cells were evaluated. The distribution of the radiotracers in rats was examined using PET imaging, and GFR values were determined and compared with inulin clearance, which is considered a reference standard. The results showed that neither radiotracer bound to blood cells; however, ^68^Ga-DTPA exhibited strong binding to plasma proteins exceeding 60% within 10 min and approaching 90% after 30 min.

In comparative studies involving inulin clearance for GFR assessment, it was found that GFR values obtained using ^68^Ga-EDTA were consistent with those measured via inulin. However, GFR values measured using ^68^Ga-DTPA were lower, falling below 80% of normal GFR values.^75^

#### ^68^Ga-1,4,7-Triazacyclononane-1,4,7-triacetic Acid (^68^Ga-NOTA)

Another hydrophilic chelating agent, NOTA, is rapidly cleared from the body via glomerular filtration. It has been labeled with both radioactive and nonradioactive tracers and utilized in kidney function tests. In recent years, its potential application in renal imaging has been explored through radiolabeling with Ga-68.

In one study, EDTA, DTPA, DOTA, and NOTA were labeled with Ga-68, and their binding affinities to serum components and red blood cells (RBCs) were assessed. Among these candidates, ^68^Ga-NOTA demonstrated the highest labeling efficiency and the lowest binding to both RBCs and plasma proteins, making it the most promising agent. As a result, PET imaging studies were conducted using ^68^Ga-NOTA and compared with those using the reference standard ^51^Cr-EDTA. Biodistribution studies showed that both radiotracers exhibited high renal uptake, and the GFR values obtained with ^68^Ga-NOTA were comparable to those obtained with ^51^Cr-EDTA. Furthermore, the GFR values derived from ^68^Ga-NOTA PET imaging were reported to closely match those determined by FITC-inulin clearance and creatinine clearance in previous studies. Based on these findings, it was concluded that ^68^Ga-NOTA is a suitable candidate for both PET-based renal imaging and accurate GFR assessment.^77^

#### ^68^Ga-1,4,7,10-Tetraazacyclododecane-1,4,7,10-tetraacetic Acid (^68^Ga-DOTA)

DOTA, which has similar pharmacokinetic properties with DTPA, has emerged as a PET radiotracer for GFR assessment. In a study conducted by Kersting et al.,^78^ PET images obtained using ^68^Ga-DOTA were reported to be of superior quality compared to conventional scintigraphy. Furthermore, the study demonstrated that noninvasive GFR measurements could be achieved using single-compartment modeling of dynamic ^68^Ga-DOTA PET data, and that these measurements showed good correlation with serum creatinine-based GFR estimates. However, despite these promising results, studies investigating this approach remain limited, and further research is needed to validate its clinical applicability.

#### ^68^Ga-*N*,*N*′-Bis­[2-hydroxy-5-(carboxyethyl)­benzyl]­ethylenediamine-*N*,*N*′-diaspartic Acid Derivative of Diacetic Acid (^68^Ga-HBED-CC-DiAsp)

^68^Ga-HBED-CC-DiAsp is one of the Ga-68 labeled renal PET radiopharmaceuticals investigated within the scope of developing agents with improved in vivo properties compared to existing renal imaging tracers. It has been reported that its binding affinity to plasma proteins and erythrocytes is similar to that of ^68^Ga-EDTA, and that it is rapidly eliminated via the renal-urinary system during PET/CT imaging. Compared to ^68^Ga-EDTA, its reported advantages include a higher stability constant and a faster chelation rate. Both in vitro and in vivo biodistribution studies suggest that this compound may possess favorable radiochemical properties for the measurement of GFR.^79^

#### ^18^F-Fluorodeoxy Sorbitol (^18^F-FDS)

^18^F-FDS is a novel PET radiopharmaceutical synthesized by reducing the aldehyde group of ^18^F-FDG a glucose analog and the most widely used PET agent in oncological applications into a hydroxyl group under appropriate conditions. Although studies on ^18^F-FDS remain limited, current research has yielded promising results in tumor, infection, and renal imaging (Figure 5).

**Figure 5:**
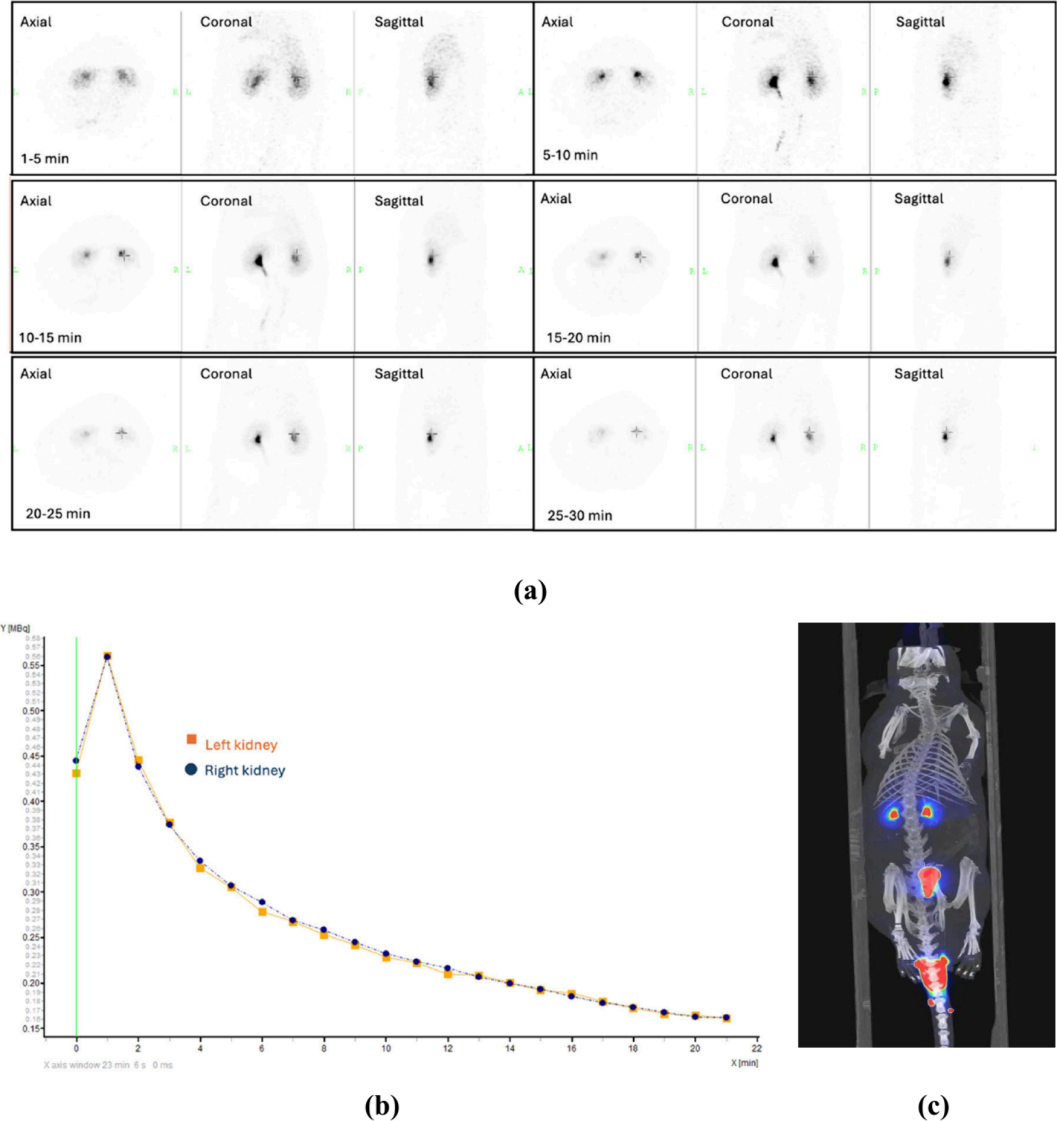
(a) Dynamic ^18^F-FDS PET images of a male Wistar rat (∼300 g) acquired using a microPET scanner, (b) time–activity curves generated from dynamic PET acquisition, and (c) whole-body ^18^F-FDS PET image obtained 30 min postinjection. (Original work of the authors.)

The first study evaluating its use in brain tumor imaging was conducted by Li et al.^80^ They demonstrated the use of ^18^F-FDS for visualizing peritumoral inflammation in a small animal model, particularly around the brain region. These encouraging results, along with the ease of synthesis from ^18^F-FDG, laid the foundation for subsequent research. In later studies, various reducing agents, reaction temperatures, and durations were tested to optimize the synthesis of ^18^F-FDS.^80–85^

In addition to its oncological applications, ^18^F-FDS has been investigated for functional renal imaging due to its inulin-like urinary excretion profile.^81^ The sorbitol structure and inulin-like kinetic behavior of ^18^F-FDS facilitate direct glomerular filtration without tubular reabsorption or secretion, allowing for accurate GFR assessment. Plasma protein binding is another crucial parameter for GFR measurement. Sorbitol was found to have a urinary clearance rate nearly identical to that of inulin (sorbitol:inulin clearance ratio = 1.01).^8,86^ Like inulin, ^18^F-FDS exhibits low binding to erythrocytes and plasma proteins, enhancing its suitability for renal function monitoring.^8^ Wakabayashi et al.^2^ reported that its plasma protein binding was less than 0.1%, further supporting its potential in GFR-based PET imaging.

Werner et al.^10^ evaluated the utility of ^18^F-FDS in functional renal imaging using rat models with acute renal failure (ARF) and unilateral ureteral obstruction (UUO). Whole-body dynamic PET imaging in healthy control animals demonstrated minimal hepatobiliary clearance and efficient renal excretion of the radiotracer. The renal cortex was clearly visualized, with a gradual transition of activity into the collecting system and renal pelvis. In ARF rats, renal cortical uptake was markedly reduced, and urinary excretion of the tracer was impaired, with delayed bladder visualization. In UUO rats, mild to moderate cortical uptake was observed on the obstructed side, with no transition to the collecting system or renal pelvis, while the nonobstructed kidney showed imaging patterns similar to healthy controls. These findings confirmed that ^18^F-FDS possesses favorable pharmacokinetics for functional renal imaging in disease models and that PET imaging offers advantages in such assessments.

The only reported clinical study involving ^18^F-FDS included two healthy human volunteers. Dynamic renal PET imaging showed initial uptake in the cortex, followed by gradual activity transfer to the parenchyma and subsequent radiotracer excretion. No adverse effects were reported. Given the intrinsic advantages of PET, ^18^F-FDS may enable a more comprehensive evaluation of renal function in humans.^87^

### 2.2 PET Radiopharmaceuticals Used for Glomerular Mesangial Function

#### ^68^Ga-IRDye800-tilmanocept

Diabetic nephropathy is one of the leading causes of kidney disease.^88^ It is characterized by albuminuria and impaired GFR.^89^ A decline in GFR typically occurs during the advanced stages of nephropathy; therefore, imaging techniques that provide reliable functional information are essential.^90^

^68^Ga-IRDye800-tilmanocept binds to glomerular mesangial cells via specific receptors, enabling the monitoring of diabetic nephropathy progression.^8^ Qin et al.^91^ emphasized the importance of monitoring mesangial cell function based on the fact that mesangial cell matrix enlargement is a histological feature of diabetic nephropathy and occurs before the patient’s glomerular filtration rate decreases, and all clinical symptoms of diabetic nephropathy are highly correlated with mesangial matrix enlargement.

In this context, the authors proposed that mesangial cell function could be monitored by targeting CD206, a receptor expressed on the surface of mesangial cells, using an appropriate radiopharmaceutical. To support this approach, they conducted a preclinical study in rats to demonstrate the receptor-mediated binding of ^68^Ga-IRDye800-tilmanocept to CD206 and to evaluate the sensitivity of this binding to changes in kidney function.

^68^Ga-tilmanocept uptake reflects increased glomerular basement membrane permeability, mesangial cell proliferation, and elevated CD206 receptor density. These changes collectively indicate the potential of this agent to detect early declines in glomerular function during the initial stages of diabetic nephropathy.

### 2.3 PET Radiopharmaceuticals Used for ERPF Assessment

#### ^11^C-*p*-Aminobenzoic Acid (^11^C-PABA)

*p*-Aminohippuric acid is considered the gold standard for measuring ERPF due to its high rate of renal tubular secretion. Therefore, ^11^C-PABA were investigated to provide reliable information regarding ERPF by PET modalities.

Ruiz-Bedoya et al.^65^ evaluated this agent for high-quality PET imaging of the kidneys in healthy rats and rabbits and conducted a comparative study with ^99m^Tc-MAG3, which is frequently used for ERPF measurements. Dynamic PET images in both healthy rats and rabbits demonstrated rapid accumulation of ^11^C-PABA in the renal cortex, followed by prompt excretion. Compared to ^99m^Tc-MAG3 in rabbits, ^11^C-PABA exhibited lower background activity in normal tissues and required a lower dose upon second administration. They also evaluated this agent in humans, and according to their findings, ^11^C-PABA was reported to be safe and well-tolerated, with no adverse or detectable pharmacological effects observed. PET imaging demonstrated initial uptake in the renal cortex, followed by a gradual progression of activity to the medulla and eventually to the renal pelvis. These findings suggest that ^11^C-PABA may serve as a novel radiotracer for functional renal imaging, offering high-quality spatial and temporal resolution with minimal radiation exposure. However, the primary limitation of ^11^C-PABA is the short half-life of carbon-11 (20 min), which necessitates the availability of an on-site cyclotron.

#### *p*-[^18^F]­Fluorohippurate (^18^F-PFH)

The molecular structure of ^18^F-PFH is similar to that of *p*-aminohippurate which is recognized as the gold standard for measuring ERPF; therefore, it is considered a potential radiopharmaceutical for renal imaging. Positive findings from the synthesis of ^18^F-PFH and subsequent studies evaluating its in vivo efficacy suggest that ^18^F-PFH is a promising renal PET radiopharmaceutical warranting further investigation.^92^

^18^F-PFH was also assessed as a PET imaging agent for the early diagnosis of polycystic kidney disease (PKD). The results indicated that the T20/T2 ratio obtained from ^18^F-PFH PET renography at an early age may serve as a novel prognostic marker for predicting the future severity of autosomal dominant polycystic kidney disease.^93^

#### Re­(CO)_3_(^18^F-FEDA)

Another radiopharmaceutical under investigation is Re­(CO)_3_(^18^F-FEDA), which reflects tubular function and ERPF. A comparison of ^99m^Tc­(CO)_3_(FEDA) with ^131^I-OIH, the standard radiopharmaceutical for ERPF measurements, demonstrated that both agents have similar properties and exhibit rapid clearance. In a subsequent study, the PET analogue, Re­(CO)_3_(^18^F-FEDA), was evaluated against ^131^I-OIH. Biodistribution studies in rats showed minimal hepatic/gastrointestinal elimination and rapid, highly specific renal extraction, comparable to that of ^131^I-OIH.^94^

#### Al-^18^F-NODA-Butyric Acid

Al-^18^F-NODA-butyric acid have been evaluated for accurate measurement of ERPF. The pharmacokinetic properties of Al-^18^F-NODA-butyric acid and ^131^I-OIH were compared in both normal rats and rats with renal failure to assess renal function. Al-^18^F-NODA demonstrated good in vitro and in vivo stability, whereas ^131^I-OIH showed higher retention in the blood due to its greater plasma protein binding. Biodistribution data indicated that Al-^18^F-NODA was excreted exclusively via the urinary system and exhibited lower hepatic and gastrointestinal activity compared to ^131^I-OIH. Furthermore, NODA was rapidly transferred from the kidneys to the bladder. Although the renal excretion of Al-^18^F-NODA was lower than that of ^131^I-OIH, the urinary activity of the two tracers was comparable in normal rats. Based on these findings, Al-^18^F-NODA-butyric acid appears to have acceptable pharmacokinetic and chemical properties as a renal PET imaging agent. Its exclusive renal excretion profile supports its potential for reliable ERPF measurement.^95^

### 2.4 PET Radiopharmaceuticals Used for Evaluation of Kidney Cancers

The diagnosis of renal masses is typically made using abdominal CT, MRI or renal mass biopsy. The major limitations of CT and MRI include their inability to reliably differentiate between benign and malignant renal lesions and their lack of capability to provide information on disease biology. Renal mass biopsy, an invasive procedure, is associated with a high nondiagnostic rate (up to 15%), low negative predictive value, and notable discrepancies with definitive histopathology.^96^

PET imaging can serve a complementary role in certain scenarios, particularly when other imaging modalities (e.g., CT, MRI) are limited. However, PET imaging with currently available tracers plays a minimal role in the diagnosis and characterization of renal tumors due to low uptake, limited specificity and sensitivity, and excretion via the urinary collecting system. Consequently, ongoing research is focused on developing alternative PET agents aimed at improving the diagnosis and evaluation of kidney cancers.

#### ^18^F-Fluorodeoxyglucose (^18^F-FDG)

PET/CT with ^18^F-FDG is the most commonly used functional imaging modality in oncology, primarily applied for staging and follow-up of malignant tumors. Because FDG is transported into cells via glucose transporters, it demonstrates high uptake in tissues with increased glucose metabolism, such as malignant and inflamed tissues, as well as physiological uptake in the brain, heart, liver, spleen, gastrointestinal tract, and bone marrow. It is also known to accumulate at high levels in the kidneys and urinary tract due to urinary excretion.^97^

^18^F-FDG is filtered through the glomerulus and partially reabsorbed in the proximal tubule, resulting in high renal uptake. Consequently, it may not be an ideal PET radiotracer for renal imaging.^8^ However, recent studies have indicated that it may be particularly useful in detecting renal cell carcinoma (RCC) metastases (Figure 6), postoperative recurrence, and RCC arising in acquired cystic disease (ACD) among patients with chronic renal failure on dialysis, where FDG excretion into the urinary tract is reduced.^98^

**Figure 6:**
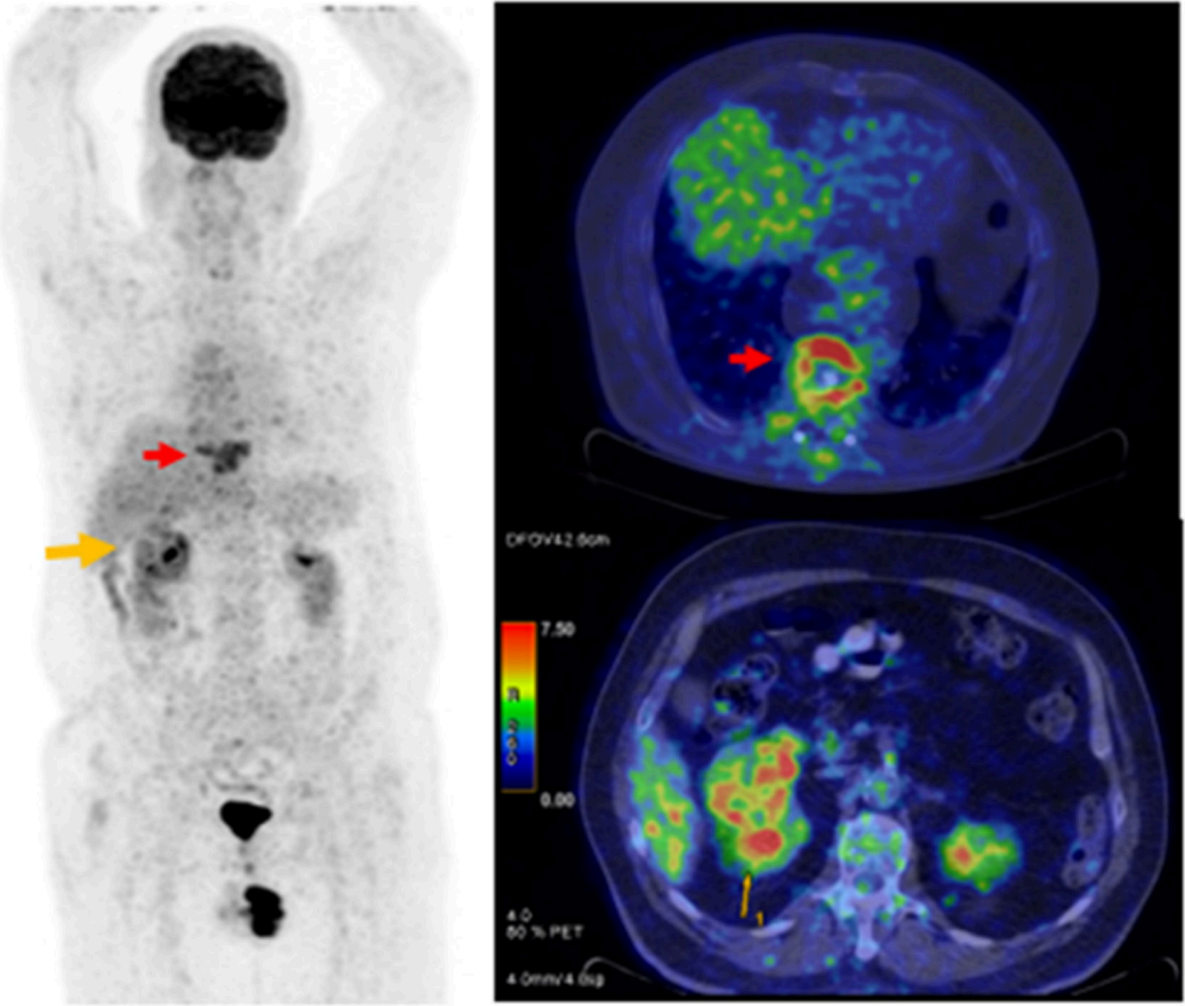
A 48-year-old male patient was evaluated for right renal mass (orange arrows), and F-18 FDG PET revealed a bone metastasis also (red arrows). The image is from the authors’ clinical database.

In addition, immune checkpoint inhibitors (ICIs) can cause autoimmune toxicities, including ICI-associated acute kidney injury (ICI-AKI). The most common histopathological finding in patients with ICI-AKI is acute tubulointerstitial nephritis (ATIN), which requires kidney biopsy for definitive diagnosis. The high prevalence of AKI among cancer patients, combined with frequent contraindications to biopsy in this population, poses a significant clinical challenge. This highlights the need for noninvasive diagnostic methods for ICI-AKI. A recent study demonstrated that ^18^F-FDG PET/CT may serve as a useful adjunctive tool for diagnosing ICI-AKI in patients with available baseline imaging, although larger prospective studies are warranted.^99^

The prognostic significance of ^18^F-FDG uptake in overall survival among patients with RCC,^100,101^ the potential value of delayed ^18^F-FDG PET/CT imaging after diuretic administration for the diagnosis of renal pelvic cancer,^102^ and its application in the assessment of renal inflammation^103–105^ have also been explored in various studies.

### 2.5 Newly Investigated PET Agents for Evaluation of Kidney Cancers

Renal cell carcinomas (RCCs), which account for approximately 2% of the annual global tumor incidence, represent nearly 90% of all solid kidney masses. Although more than 12 histological subtypes have been identified, clear cell renal cell carcinoma (ccRCC) is the most common and aggressive form of kidney cancer. Small renal masses are frequently detected incidentally during abdominal imaging, with incidence rates rising due to population aging and the global obesity epidemic. Furthermore, approximately 20–30% of patients are at risk of metastatic recurrence following treatment.

^18^F-FDG PET/CT, a widely used whole-body tumor imaging modality, has shown limited performance in detecting primary ccRCC lesions. Reported sensitivity ranges from 63.6% to 90.0%, with reduced accuracy primarily attributed to high physiological background activity and inflammation.^106^ This underscores the need for more specific diagnostic and imaging tools for RCC, particularly ccRCC.

#### Carbonic Anhydrase IX (CAIX) as a Molecular Target in ccRCC

Carbonic anhydrase IX (CAIX) is a cell membrane-bound enzyme that is upregulated under hypoxic conditions and plays a key role in tumor cell survival and metastasis. While minimally expressed in normal tissues, CAIX is highly expressed in certain tumors. Approximately 80–90% of patients with ccRCC carry mutations in the von Hippel–Lindau (VHL) tumor suppressor gene, leading to stabilization of hypoxia-inducible transcription factors (HIF-1α, HIF-2α) and subsequent overexpression of target genes, including CAIX. Notably, CAIX is expressed in over 90% of ccRCC cases, whereas its expression in other RCC subtypes (e.g., papillary RCC, chromophobe RCC) is minimal or absent, making it an attractive target for ccRCC imaging and therapy.

The first CAIX-targeting study utilized girentuximab, a mouse IgG1 monoclonal antibody, initially radiolabeled with iodine^107^ and later with indium-111 (In-111)^108^ and zirconium-89 (Zr-89).^109^ Results from a Phase III clinical trial involving approximately 284 patients demonstrated that [^89^Zr]­Zr-girentuximab had a favorable safety profile and provided highly accurate, noninvasive detection and characterization of ccRCC, with the potential to change clinical practice.^96^

Recognizing the limited tumor penetration and slow blood clearance of large-molecule antibodies, Lou et al.^106^ developed NYM046, a novel small-molecule acetazolamide-based compound targeting CAIX. Labeled with Ga-68 using DOTA as a chelator, [^68^Ga]­Ga-NYM046 was first evaluated in a xenograft tumor model and subsequently in patients with ccRCC. A total of 47 patients (mean age, 58.8 ± 13.5 years; 34 males) were enrolled in the study and divided into two groups. Group 1 consisted of 20 patients with primary renal masses scheduled for surgery, while Group 2 included patients with suspected or confirmed metastatic ccRCC. Clinical results revealed high renal uptake due to urinary excretion and high gastric accumulation attributable to physiological CAIX expression in the gastric mucosa. The sensitivity, specificity, and accuracy of [^68^Ga]­Ga-NY104 PET imaging were 62%, 33%, and 58% for Group 1, and 95%, 100%, and 96% for Group 2, respectively. [^68^Ga]­Ga-NY104 PET identified 26 additional disease sites in 67% of the patients (14/21) that had not been previously recognized, and the tumor uptake was found to correlate with immunohistochemical staining results. A positive correlation between CAIX expression and SUVmax was observed, supporting the potential of CAIX-targeted tracers for broad clinical application in ccRCC, although further validation is warranted.^106,110^

#### CD70 as an Emerging Target

Another promising molecular target for ccRCC is CD70, a TNF superfamily surface molecule typically expressed transiently on antigen-presenting cells, including B cells, T cells, and dendritic cells. CD70 regulates immune responses—such as T-cell activation, proliferation, and memory formation—via its receptor CD27. CD70 is overexpressed in approximately 70–80% of ccRCC cases but is absent or expressed at low levels in normal renal tissue and other RCC subtypes.

Wu et al.^111^ developed four CD70-specific single-domain antibodies (sdAbs) labeled with F-18. Based on the results obtained from their in vitro and in vivo studies one candidate, ^18^F-RCCB6, was selected and applied to six patients (age range: 58–77 years; five males and one female) with histologically confirmed RCC. All patients underwent PET/CT imaging from the head to the midthigh region 1 h after radiopharmaceutical administration. Based on the results obtained, the researchers reported that immune PET/CT imaging with [^18^F]­RCCB6 was a safe procedure and that this tracer exhibited high uptake in tumor tissue while maintaining low background signal in organs outside the urinary tract. Metastatic ccRCC lesions in the lungs, bones, pancreas, muscle, lymph nodes, and intracranial (endocranial) regions were clearly identified. ^18^F-RCCB6 immuno PET/CT identified ccRCC metastases in multiple patients and demonstrated superior contrast and diagnostic performance compared with ^18^F-FDG PET/CT in at least one case. Lesion-based analyses demonstrated that [^18^F]­RCCB6 uptake was generally higher than that of ^18^F-FDG (*P* = 0.035). However, the authors emphasized that the relationship between [^18^F]­RCCB6 imaging parameters and CD70 expression in tumor lesions should be further investigated in larger study cohorts.

#### Prostate-Specific Membrane Antigen (PSMA) as a Theranostic Target in RCC

Prostate-specific membrane antigen (PSMA) is a type II transmembrane glycoprotein encoded by the folate hydrolase (FOLH1) gene. Initially identified as a target for the monoclonal antibody 7E11-C5.3 in prostate cancer cell lines,^112^ PSMA has since become an established target for both imaging (staging and therapy response evaluation) and radionuclide therapy in prostate cancer. Although strongly associated with prostate malignancies, PSMA is also overexpressed in the microvasculature of numerous solid tumors, including RCC. This has prompted investigation into its diagnostic and therapeutic potential in RCC.

The first reported use of ^68^Ga-PSMA PET/CT for diagnosing metastatic ccRCC was described by Demirci et al.,^113^ who found it detected significantly more metastatic lesions compared with ^18^F-FDG PET/CT.

In a retrospective analysis of 257 RCC patients, including clear cell, papillary, and chromophobe subtypes, Spatz et al.^114^ reported that stronger PSMA expression correlated with higher tumor grade, more advanced stage, and worse overall survival. Furthermore, ^18^F-DCFPyL, a PSMA-targeted PET tracer used in prostate cancer, demonstrated higher sensitivity for small lesion detection and greater uptake in metastatic RCC compared with ^18^F-FDG in head-to-head studies.^115,116^

While PSMA-targeted tracers show promise in RCC imaging, challenges remain. In localized disease, low tumor-to-background ratios limit local staging accuracy. Novel tracers with reduced or absent renal excretion, such as PSMA-1007, may help overcome these limitations and improve clinical utility.^117^

#### Fibroblast Activation Protein Inhibitors (FAPIs) as a Theranostic Target in RCC

Cancer-associated fibroblasts (CAFs) are abundantly present in tumor microenvironments, where they regulate immune responses and promote tumor growth, cell invasion, and metastasis. In these regions, fibroblast activation protein (FAP) is markedly overexpressed. In recent years, radiolabeled fibroblast activation protein inhibitors (FAPIs) have been extensively investigated as molecular PET/CT imaging agents in various tumor types, particularly in those exhibiting low or absent ^18^F-FDG uptake. Unlike ^18^F-FDG, ^68^Ga-FAPI imaging does not require fasting or dietary preparation, which represents an additional practical advantage. Accordingly, several FAPI derivatives have been explored for their diagnostic performance in various tumor types.

Although ^68^Ga-labeled FAPI compounds are more frequently utilized, the relatively short half-life of Ga-68 is considered a limitation, prompting interest in developing F-18 labeled FAPI analogs as alternatives. The first study in this field, conducted by Giesel et al., compared [^18^F]­F-AlF-FAPI-74 and [^68^Ga]­Ga-FAPI-74 in patients with lung cancer in terms of radiation dosimetry, tumor delineation, and biodistribution.^118^ Subsequent studies have reported that the whole-body effective dose of [^18^F]­F-FAPI is lower than that of ^68^Ga-labeled FAPI probes. Various preclinical and clinical investigations have demonstrated high tumor uptake and favorable tumor-to-background ratios in multiple tumor types using [^18^F]­F-FAPI PET/CT.^119–122^

Renal cancer is one of the various tumor types in which the use of radiolabeled FAPIs has been investigated. In a systematic study by Pandey et al., comparing ^68^Ga-FAPI PET/CT with ^18^F-FDG in renal cancers, ^68^Ga-FAPI PET/CT demonstrated superior tumor-to-background ratios and was more effective in detecting small primary or metastatic lesions that were often missed by other radiotracers. Moreover, ^68^Ga-FAPI showed rapid and high tumor uptake, allowing lesion visualization as early as 10 min postinjection. The tracer exhibited very low background uptake in the renal cortex, facilitating primary lesion detection, and provided high image contrast in the brain and abdominal regions due to slower physiological uptake compared to ^18^F-FDG.^123^

In a prospective study involving 11 patients with fumarate hydratase-deficient renal cell carcinoma (FHRCC) encompassing 83 lesions, ^68^Ga-FAPI-04 PET/CT and ^18^F-FDG PET/CT were compared. Although ^18^F-FDG PET/CT showed higher lesion detection rates than ^68^Ga-FAPI-04 PET/CT (primary tumors: 75.0% vs 50.0%; lymph nodes: 94.9% vs 89.7%; bone lesions: 100.0% vs 90.5%), semiquantitative analysis revealed comparable median SUVmax values between the two tracers (primary lesions: 13.86 vs 16.35, *P* = 1.000; lymph nodes: 10.04 vs 9.33, *P* = 0.517; bone lesions: 13.49 vs 9.84, *P* = 0.107; visceral lesions: 8.54 vs 4.20, *P* = 0.056). Nevertheless, median tumor-to-liver ratios (TLR) for ^68^Ga-FAPI-04 PET/CT were significantly higher than those for ^18^F-FDG PET/CT (primary lesions: 30.44 vs 5.41, *P* = 0.010; lymph nodes: 17.71 vs 3.95, *P* < 0.001; bone lesions: 15.94 vs 5.21, *P* < 0.001; visceral lesions: 9.26 vs 3.44, *P* = 0.003). These elevated TLR values suggest a promising theranostic potential for radiolabeled FAPI tracers in renal malignancies.^124^

## 3.0 Future Directions

In recent years, research on renal radiopharmaceuticals has increasingly focused on PET-based agents. Future investigations in this field are expected to continue toward the development of novel radiopharmaceuticals that not only serve as diagnostic imaging tools but also enable a comprehensive assessment of renal structure and function.

In addition to cancer, many nonmalignant renal diseases can affect both the structure and function of the kidneys, leading to a variety of clinical complications. PET/CT holds great potential as a versatile modality for evaluating such conditions. The widely used oncologic tracer ^18^F-FDG has been shown to provide valuable complementary information for the diagnosis and treatment selection of various nononcologic renal diseases including acute pyelonephritis, immune complex mediated glomerulonephritis, chronic kidney disease, renal histiocytosis, and renal amyloidosis.^125^

Similarly, radiolabeled FAP inhibitors (FAPIs), initially developed for oncologic imaging, have shown highly promising results in the diagnosis of several nonmalignant diseases. In addition to its overexpression in cancer-associated fibroblasts (CAFs) present in most epithelial tumors, FAP can also be expressed during extracellular matrix remodeling and therefore FAP has been detected in various benign pathological processes such as wound healing, chronic inflammation, arthritis, fibrosis, and ischemic cardiac tissue following myocardial infarction. Because of their high affinity for both malignant and benign fibroblast-rich processes, radiolabeled FAPIs have been also proposed as potential diagnostic tools for nononcologic diseases, providing not only diagnostic insights but also valuable information regarding the role of the tissue microenvironment.^126^ Several studies have demonstrated the use of Ga-68 and F-18 labeled FAPIs for imaging renal fibrosis occurring in a range of pathological conditions, including maladaptive repair leading to organ fibrosis after acute kidney injury,^127^ renal fibrosis associated with acute rejection following kidney transplantation,^128^ hypertension-induced renal dysfunction characterized by proteinuria and decreased glomerular filtration rate progressing to chronic kidney disease,^129^ and active renal tubulointerstitial fibrosis in patients with lupus nephritis.^130^

Beyond their diagnostic capabilities, FAPI-based radiopharmaceuticals are also being actively investigated for therapeutic applications under the concept of FAPI radioligand therapy (FAPI-RLT).^131^

Although current evidence suggests that these agents represent a promising and safe diagnostic and/or therapeutic option for various renal diseases in the short term, further large-scale and controlled clinical studies are required to evaluate their long-term efficacy, safety, and clinical utility.

As in many other areas, the integration of PET imaging with artificial intelligence–driven quantitative image analysis is expected to become a major focus of future research in renal imaging, aiming to enhance the accuracy of quantitative assessment.^132,133^

Collectively, these advances may enable earlier disease detection and more precise monitoring of therapeutic response. The incorporation of PET-based functional imaging into nephrology practice could allow for more detailed disease characterization, contribute to the optimization of treatment planning, and significantly support the advancement of precision medicine in renal care.

## Conclusion

The determination and imaging of the structure, morphology, pathophysiology, and function of the kidneys are of great importance in the diagnosis of kidney diseases, in monitoring treatment, and in the evaluation of other kidney-related disorders. Nuclear medicine has provided significant advantages in renal imaging for many years. In addition to enabling the assessment of split renal functional parameters such as GFR, ERPF, tubular function, and renal blood flow, it also facilitates the evaluation of various pathophysiological processes within the renal parenchyma.

Although conventional renal scintigraphy radiopharmaceuticals are still used in clinical practice, recent advances in nuclear medicine have transformed the landscape of renal functional imaging, providing more sensitive, quantitative, and molecularly specific insights than conventional modalities. Among these, PET radiopharmaceuticals have emerged as powerful tools for assessing glomerular filtration rate, tubular secretion, plasma flow, and metabolic activity with high spatial and temporal resolution. Novel tracers such as Ga-68 and F-18 labeled compounds have demonstrated strong potential in preclinical and early clinical studies, offering the possibility of more accurate diagnosis and individualized patient management. Despite these promising developments, most renal PET tracers remain in the research phase, and further validation through clinical trials is required before routine implementation.
